# Multidomain interventions for non-pharmacological enhancement (MINE) program in Chinese older adults with mild cognitive impairment: a multicenter randomized controlled trial protocol

**DOI:** 10.1186/s12883-023-03390-5

**Published:** 2023-09-28

**Authors:** Xiaochu Wu, Tianyao Zhang, Yanhao Tu, Xueling Deng, A Sigen, Yuxiao Li, Xiaofan Jing, Lixuan Wei, Ning Huang, Ying Cheng, Linghui Deng, Shuli Jia, Jun Li, Ning Jiang, Birong Dong

**Affiliations:** 1 National Clinical Research Center for Geriatrics, West China Hospital of Sichuan University, Chengdu, China; 2 The First Affiliated Hospital of Chengdu Medical College, Chengdu, China; 3 Strength and Conditioning Center, Chengdu Sport University, Chengdu, China

**Keywords:** Mild cognitive impairment, Randomized Controlled Trial, Multidomain Interventions, Non-pharmacological, Older adults

## Abstract

**Background:**

Dementia is characterized by progressive neurodegeneration and therefore early intervention could have the best chance of preserving brain health. There are significant differences in health awareness, living customs, and daily behaviors among Chinese older adults compared to Europeans and Americans. Because the synergistic benefits of multidomain non-pharmacological interventions are consistent with the multifactorial pathogenicity of MCI, such interventions are more appealing, easier to adhere to, and more relevant to daily life than single-mode interventions. One of the aims of this study is to verify the effect of multidomain intervention strategies for MCI patients based on Chinese population characteristics, and the other is to establish a biobank and image database to investigate the pathogenesis and pathways of cognitive impairment.

**Methods:**

Our study was designed as a national multicenter, community-based randomized controlled trial (RCT). Twelve medical institutions in ten Chinese cities will participate in our study from 2020 to 2024, and 1080 community residents aged 50 and above will be enrolled as participants. Each sub-center will be responsible for 90 participants (30 people per community) across three communities (non-contact control group, health education group, and multidomain intervention group). The community will be the basic unit of the present study, and all participants in each community will receive the same intervention/control measure. Three working groups are set up in each sub-center to manage the three communities independently to minimize interference at the implementation level between the groups. The multidomain intervention group will receive integrated interventions including exercise, nutrition, sleep, health education and mindfulness meditation. All data generated by the research will be analyzed and processed by statistical software (such as SPSS 21.0, Python 3.0, etc.), and part of the research data will be displayed in the form of graphs and tables.

**Discussion:**

In order to achieve a high-quality community intervention study, it is crucial to have a well-designed experimental protocol that follows rigorous scientific methodology. In addition, effective management of quality control measures and monitoring compliance throughout the study process are essential components. This study provides a detailed discussion of stakeholder compliance, research quality control, potential harm and mitigation, auditing, and future plans in order to better address research issues.

**Trial registration:**

: ChiCTR2000035012 (July 27, 2020).

**Supplementary Information:**

The online version contains supplementary material available at 10.1186/s12883-023-03390-5.

## Background

Dementia is a clinical syndrome characterized by chronic, acquired, and progressive mental decline and memory loss, resulting in an inability to perform daily activities. Alzheimer’s disease (AD) is the most common, accounting for 60–80% of all dementia cases [1].

World Health Organization (WHO) statistics indicate that a new AD patient is diagnosed every three seconds globally. According to the World Alzheimer’s Disease Report 2019, approximately 50 million people are suffering from AD around the globe. This number is expected to rise to 152 million by 2050, with an annual global economic burden of approximately one trillion US dollars [2].

China has the highest number of dementia patients in the world. According to the “Healthy China Action (2019–2030)” released in 2019, the prevalence rate of AD among people aged 65 and above is 5.56%, accounting for 20% of global patients, up to 10 million, ranking first in the world. By 2050, it is expected to exceed 40 million [3].

The course of AD is obscure, and it is not easy to diagnose at an early stage. Concurrently, the exact etiology and pathogenesis of the disease are still unknown, and treatment methods are still being explored. Therefore, early prevention, diagnosis, and intervention have emerged as the primary focus of global research [4]. The recent Lancet Commission on Neurology states that action to prevent dementia must be taken immediately. The latest research knowledge and effective prevention programs must be implemented without delay. Meanwhile, the Action Plan for Healthy China (2019–2030) has clearly stated the goal of controlling the future growth rate of dementia prevalence [5].

Mild cognitive impairment (MCI) is an intermediate stage between normal aging and dementia. The latest evidence reveals that the prevalence of MCI among older adults aged 60–64 years has increased from 6.7 to 25.2% among older adults aged 80–84 [6]. However, the average prevalence of MCI in China is approximately 20.8% [7]. After three years, up to 44% of MCI patients can be converted into AD, which is ten times higher than the general population, and the probability of developing AD within five years is as high as 70%, which is the main factor for the occurrence of AD [3]. Therefore, there is a “time window” for intervention to delay disease progression and treatment. Existing evidence indicates that intervention at this stage can effectively slow or prevent MCI from progressing to dementia [8], which significantly impacts promoting healthy aging in China.

At present, some research evidence suggests that single intervention such as exercise, nutrition, sleep and meditation had a positive effect on promoting cognitive function.

### Exercise and MCI

The latest American Academy of Neurology (AAN) guidelines [9] believe that regular exercise in MCI patients can help improve cognitive function, which is recommended as part of the overall treatment. The guideline recommendation is (level B) that exercise twice a week for six months may help improve memory in people with mild cognitive impairment [9]. A network meta-analysis of 71 studies compared the effects of various types of exercise (aerobic exercise, resistance exercise, compound exercise, mind-body exercise, whole-body vibration, and finger exercises) on overall cognitive function and specific dimensions of cognitive function in patients with MCI and dementia. The optimal types of exercise interventions for MCI and dementia patients with different dimensions of cognitive function were revealed, providing evidence support for the formulation of exercise prescription and the implementation of individualized exercise intervention [10].

### Nutrition and MCI

Pathology, heredity, environment, and living habits affect cognitive function in aging, and dietary intake in living habits is one of the hot research areas in recent years. Numerous epidemiological studies reveal that diet is related to cognitive function and that healthy eating habits protect cognitive function [11]. Currently, studies on the diet of older adults with cognitive dysfunction are being conducted. Substantial evidence supports that changing diet and nutrition can help maintain good cognitive health, mood, immune function, and vascular status. The earlier the change, the better the effect. The Mediterranean-Dash diet Intervention for Neurodegenerative Delay (MIND) studies demonstrated that the MIND diet pattern could reduce the risk of AD and are more effective than the Mediterranean diet alone in slowing cognitive decline and promoting brain health [12, 13]. A follow-up study in 2015 revealed that the brain age of older adults who adopted the MIND diet was 7.5 years younger on average than the control group [14].

### Sleep and MCI

Studies demonstrated that patients with MCI have impaired sleep-dependent memory consolidation, as the dentate gyrus volume shrinks in the MCI group compared with the control group. In addition, the duration of sleep spindles is shorter, implying that the duration of sleep spindles may be related to memory consolidation. A cross-sectional study of 4,417 older adults indicated that higher beta-amyloid deposition was associated with shorter sleep duration, and both shorter (< 6 h) and longer (> 9 h) sleep duration was associated with cognitive decline and severe depression [15]. The previous study of this research team revealed that 43.8% of older adults with MCI in the community had sleep disorders.

### Meditation and MCI

Mindfulness meditation can help patients with MCI as a non-pharmacological treatment that is safe and effective. A study revealed that participants who completed a mindfulness-based meditation stress reduction program improved on measures of cognitive function and well-being, as well as positive changes in the hippocampus and other brain regions associated with cognitive decline. Most people who practice mindfulness meditation can master the fundamental principles of mindfulness, implying that memory impairment with MCI does not preclude learning this skill and that adults with MCI can improve their cognitive reserve by learning mindfulness meditation [16].

Multidimensional interventions are those that have two or more dimensions. Because the synergistic benefits generated by multidomain interventions are consistent with the multifactorial nature of MCI, international studies demonstrated that multidomain interventions have more significant benefits in improving cognitive impairment than cognitive training or exercise alone, such as several large multidomain RCTs of lifestyle in Europe Finnish Geriatric Intervention Study to Prevent Cognitive Impairment and Disability (FINGER; NCT01041989) trial in Finland, The French Multidomain Alzheimer Preventive Trial (MAPT; NCT00672685) and the Dutch Prevention of Dementia by Intensive Vascular Care (Pre-DIVA; ISRCTN29711771) trial, both of which revealed that after intervention in multiple fields such as diet, exercise, sleep, cognitive training, and vascular risk monitoring, there was a significant improvement in cognition [17, 18]. For example, the FINGER experiment in Finland indicated that the executive function and processing speed of the intervention group increased by 83% and 150%, respectively. In addition, there were significant intervention effects on other secondary outcomes such as body mass index (BMI), eating habits, and physical activity. The improvement of primary and secondary cognitive outcomes increased by 25–150% compared to the control group.

Compared with single intervention, the multi-domain intervention subjects have higher compliance, easier to adhere to, and more interactivity. However, compared with Europeans and North Americans, there are differences in protein intake, frequency of exercise, cooking/eating patterns, personal hobbies and other behaviors and lifestyles among older Asians [19–22]. There is currently insufficient evidence of early multidomain intervention studies for the older adult MCI population in Asia, particularly in Southeast Asia.

### Explanation for choice of comparators

The main aim of this study is to evaluate the effectiveness of multidomain cognitive intervention. Subjects in the non-contact control group (without any intervention) will be selected as the control group to compare the effect evaluation of different intervention intensification (multi-domain collaborative intervention and traditional health education).

### Objectives

1. A randomized controlled trial will be used to investigate and validate a multidimensional intervention strategy for MCI patients based on Chinese population characteristics.
2. To build a community non-drug multidimensional intervention biobank for MCI patients and to investigate the disease mechanism and cognitive impairment pathway.

## Methods

### Trial design and study setting

The present study was designed as a multicenter, national, community-based RCT (2020–2024). We followed the Standard Protocol Items: Recommendations for Interventional Trials guidelines (SPIRIT 2013) in developing this protocol.

This program was led by the West China Hospital of Sichuan University (WCHSCU). We advertised for research partners on the National Clinical Research Center for Geriatrics (NCRCG) platform, and 27 hospitals responded. After one-on-one communication, we decided that the project would be carried out simultaneously with 12 sub-center research partners in 10 major Chinese cities. The present study will be conducted in Chengdu City (WCHSCU, Chengdu Fifth People’s Hospital, and The First Veterans hospital of Sichuan Province), Daqing City (Daqing Long Nan Hospital), Nanning City (The First People’s Hospital of Nanning), Hangzhou City (The First Affiliated Hospital, Zhejiang University School of Medicine), Lanzhou City (The First Hospital of Lanzhou University), Nanjing City (Jiangsu Province Geriatric Hospital), Yibin City (The Second People’s Hospital of Yibin), Changchun City (Jilin Provincial People’s Hospital), Qingdao City (Qingdao Municipal Hospital), Tai’an City (Taian City Central Hospital). The signing of study cooperation agreements between WCHSCU and each sub-center and ethical application in each sub-center is in progress. As of May 2023, eight of the 12 sub-centers had completed ethical applications and signed research cooperation agreements, while the remaining four partners are still in the process.

In each sub-center city, three communities are selected: the non-contact control group, the health education group, and the multidimensional intervention group. According to the eligibility criteria, 30 participants from each community were selected to participate in the study. All participants from the same community will receive the same intervention, and all participants will participate in the study for a period of six months, the difference is that participants in different communities receive different interventions.

### Participants eligibility criteria and timeline

All participants in the present study are from natural population communities, all intervention staff must pass the uniform training and examination organized by the NCRCG before carrying out the specific intervention work.

In order to find MCI subjects in the community more accurately and quickly, our team set the following subject qualification criteria in accordance with “2018 Chinese Guidelines for Diagnosis and Treatment of Dementia and Cognitive Impairment (V): Diagnosis and Treatment of Mild Cognitive Impairment“ [23]. The following screening eligibility criteria are used:

#### Inclusion criteria

1. The participants were community residents aged 50 and above (age is subject to ID card).
2. The chief complaint of forgetfulness (self-reported memory deterioration in the last five years) or memory deterioration confirmed by a researcher.
3. The baseline Montreal Cognitive Assessment (MoCA) score of less than 26/30 are used primarily to include subjects with cognitive dysfunction; Mini-mental Status Examination (MMSE) scores greater than 22 are primarily used to include subjects with non-severe dementia [24].
4. On the daily instrumental activity scale, Lawton and Brody scored more than 6/8.
5. Being able to live in their own homes rather than hospitals or long-term care facilities.
6. Can read and write by themselves.
7. Do not take anti-dementia medications (e.g., donepezil, galantamine) recently or during the study period.
8. Sign the informed consent form, confirming that you have been informed of all relevant contents of the trial.
9. Able to walk four meters independently with or without a walker.
10. Physical Activity Readiness Questionnaire (PAR-Q) scale confirmed that the participants were healthy enough to participate in the relevant exercise.
11. By utilizing the multi-nutritional assessment short form (MNA-SF) questionnaire to assess nutritional status and also understanding the subject’s history of food allergies, clinical nutrition experts evaluate if they are eligible for nutritional intervention.
12. The Pittsburgh Sleep Quality Index (PSQI) showed no serious sleep disorder.
13. Previous medical history investigations indicate no serious mental illness and no current psychological or psychiatric treatment.
14. Complete all assessment contents and sign the informed consent form.

#### Exclusion criteria

1. In the past three months, engaged in moderate-intensity physical exercise for physical training for more than 60 min per week;
2. Diagnosed with any type of dementia.
3. Impaired cognitive function caused by neurodegenerative diseases (e.g., multiple sclerosis, Parkinson’s disease, Huntington’s disease, frontotemporal dementia) but not AD and vascular cognitive impairment.
4. Have a high risk of adverse cardiovascular events during exercise, or fail to adjust their activities according to the guidance of rehabilitation exercise experts, or achieve the recommended activity level through understanding.
5. Diagnosis of severe peripheral neuropathy or severe musculoskeletal or joint diseases that impair mobility.
6. Using medications that may impair cognitive function, such as anticholinergics, including drugs with significant anticholinergic properties (e.g., amitriptyline), major sedatives (i.e., typical and atypical antipsychotics), and anticonvulsants (e.g., gabapentin, and valproate).
7. Any hormone therapy (estrogen, progesterone, or testosterone) received within the previous 24 months.
8. Plan to or have participated in concurrent clinical drug or exercise trials.
9. The older adult with metal in the body, such as dentures (not to be removed), cardiac pacemakers, artificial hip joints, steel plates, steel nails, stents, and other metals, so that the Functional Magnetic Resonance Imaging (MRI) test results are not affected.
10. Community residents diagnosed with depression or delirium by a medical institution will not be able to participate in this study.

### Participant timeline

**Fig. 1:**
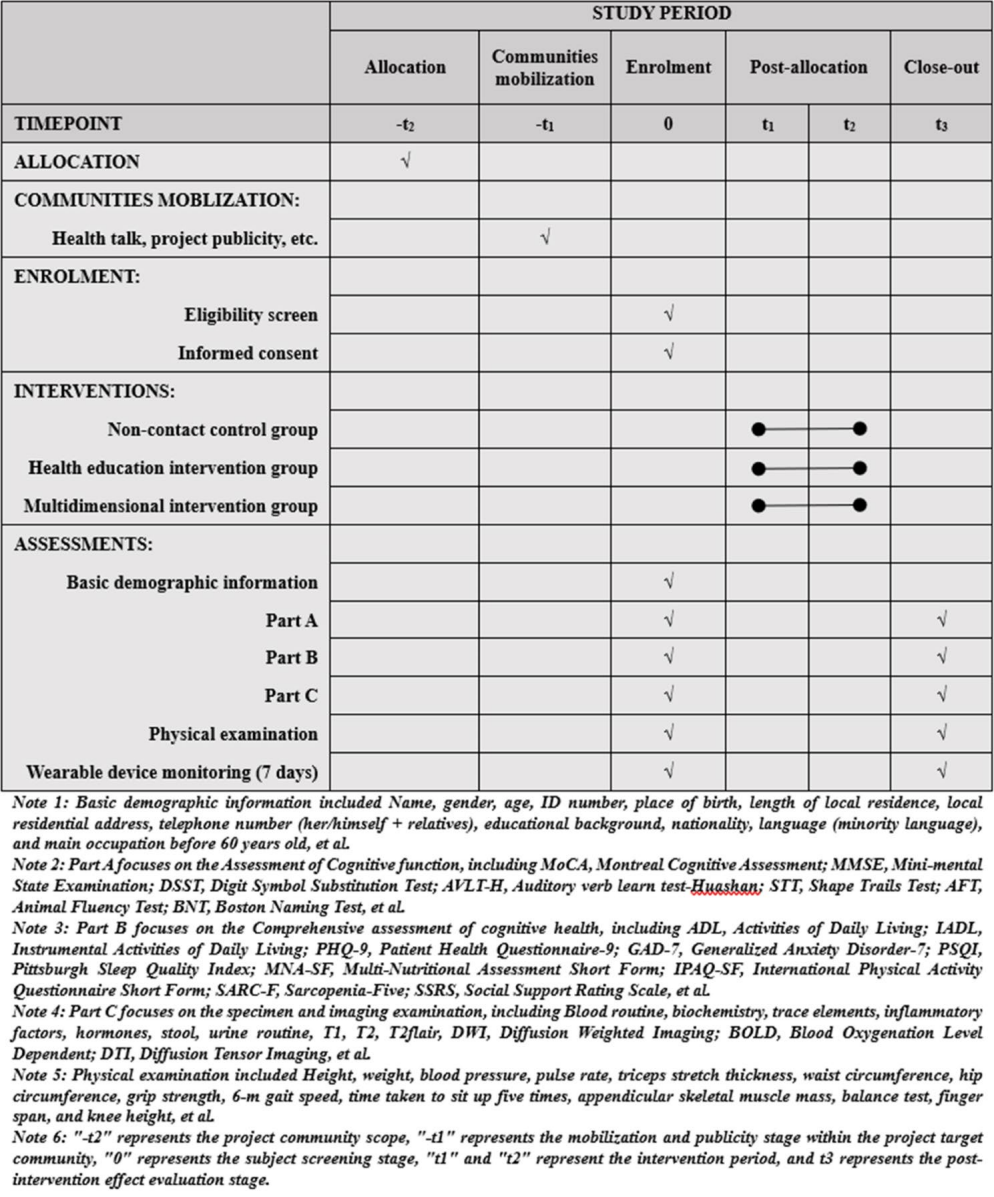
Study timeline

**Fig. 2:**
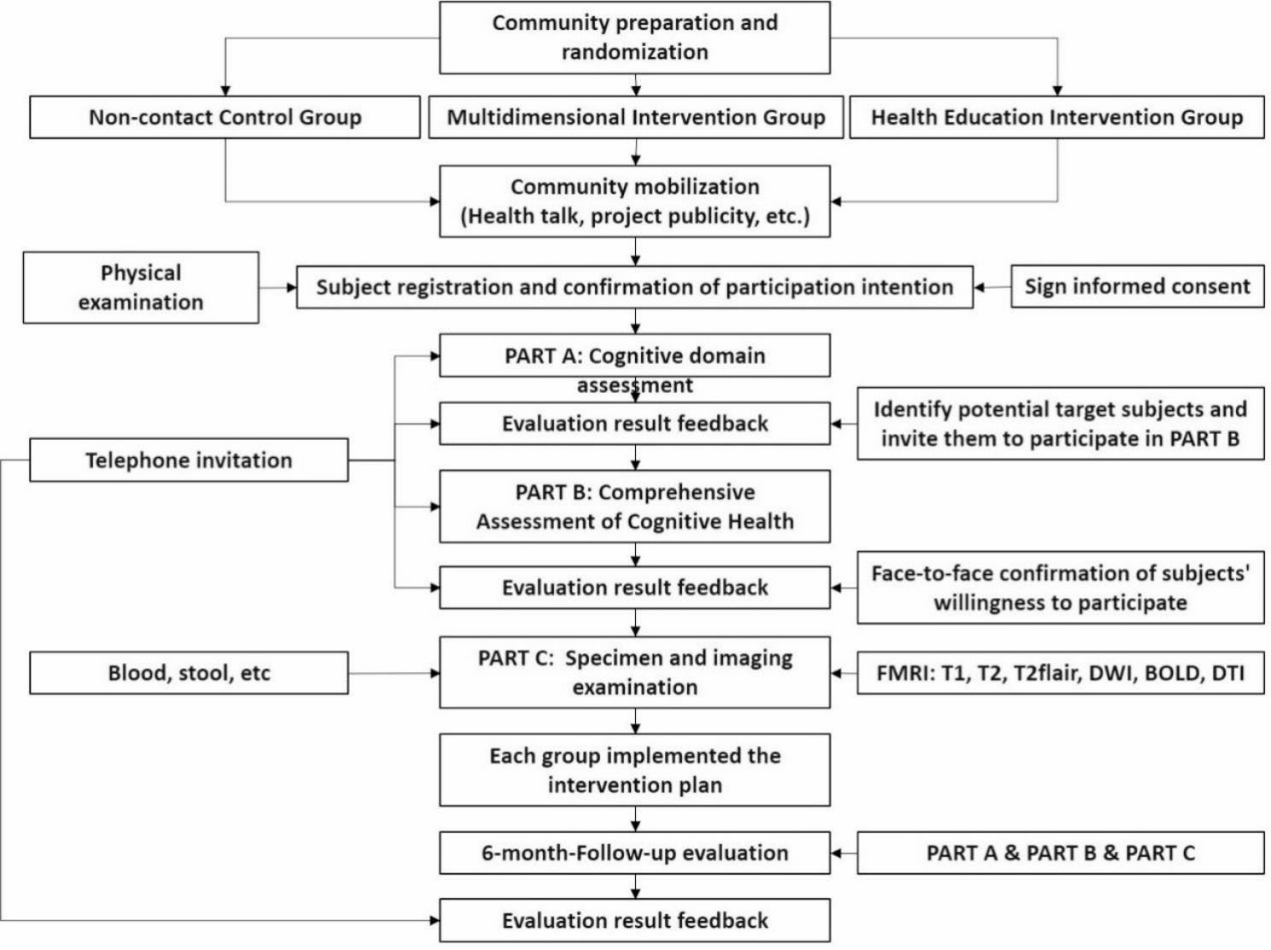
Flow of participant

Details of the study timeline and flow of participant are shown in Figs. 1 and 2.

Meanwhile, the overall arrangement of trial timeline is as follows:

2020.1-2022.12: Design, program demonstration, preliminary preparation and pilot study.

2023.2-2024.12: Conduct the formal national multicenter study.

### Sample size and groups

In the present study, the sample size is determined primarily by the following factors: [1] It is difficult to maintain the compliance of the community population, which necessitates a significant investment of workforce by the research team to maintain compliance; [2] The quality control difficulty of multidomain intervention is high, and more human and material resources required in supervising the quality of each link of the research; [3] Screening of potential subjects in the community requires high time and cost; [4] Due to the reasons of research content and funding, the number of our team members is limited; [5] MCI prevalence was set as P = 0.34 [25], power = 0.95, and dropout rate = 0.2. Therefore, considering scientific and practical feasibility and other factors, according to the sample size calculation method of RCT study [26], the total sample size of the single center would be 90, with 30 participants in each community. We have given the basis for calculating the sample size of a single sub-center. However, because China has a large geographical area, each region may have different characteristics. Therefore, in the study design, we calculated the sample size required by only one sub-center, in order to balance the influence of different regional bias factors on the overall effect of the study by adding sub-centers in different regions into the overall study, so as to evaluate the effect of multi-domain intervention more objectively. Therefore, this study intends to include 12 sub-centers to participate, with a total sample size of 1080.

### Recruitment and screening

Considering the difficulty and high dropout rate of community intervention studies, this study split all assessment/examination content of subjects into 4 steps, recruited a sufficient number of potential subjects at one time to enter the step 1, and then assessed participants’ compliance through repeated assessments of participants (steps 2 to 4, with 2–5 days intervals between each step). Finally, subjects with strong enough adherence are screened for formal study. The following are the main 4 steps in the participant screening process:

1. The research team disseminates the program in the community and compiles a list of potential participants by encouraging community residents to sign up.
2. Potential participants who meet the eligibility criteria will participate in a preliminary screening for the risk of cognitive impairment (with a sample size of more than 100 people in each community). The assessment content will mainly focus on a special assessment of cognitive domains. This assessment time will be approximately 30–40 min for each person. Participants who complete this step are considered to have passed the first compliance test.
3. A team of doctors is organized to provide community on-site answers to the preliminary screening results in the community. The potential participants are invited to participate in the cognitively related risk factors (such as the ability to live daily, anxiety, and depression). Subjects who successfully completed this step have passed the second compliance assessment.
4. The community research manager will call the potential participants (after two rounds of compliance assessment), invite them to take part in biological sample testing and functional MRI under the condition of obtaining their consent and signing the ethical, informed consent, and finally determine the list of participants.

The recruitment standardization of each participant must go through three rounds of quality control work before he or she formally enters the study:

1. The data platform manager screened out the list of eligible participants based on the assessment results recorded in the database and submitted it to the PI for the second round of review;
2. The principal investigator (PI) reviewed the eligibility conditions of the participants in the list again based on eligibility criteria and submitted the list to the community manager for the third round of confirmation;
3. Community managers conducted community on-site interviews with the community participants, informed them about the content of the informed consent again, and signed the informed consent will be by the participants themselves.

### Interventions

Our study will be mainly composed of Multidimensional intervention group, Health education intervention group and non-contact control group. The implementation scenarios of all research processes (except MRI) under this research project will be completed in the community. Each study group receives varying levels of cognitive health risk factor intervention, as presented in Table 1.

**Table 1:** Interventions and groups setting

Section	Non-contact control group	Health Education Intervention	Multidimensional Intervention
Exercise intervention	**×**	**×**	**√**
Sleep intervention	**×**	**×**	**√**
Meditation intervention	**×**	**×**	**√**
Dietary intervention	**×**	**×**	**√**
Health education intervention	**×**	**√**	**√**

#### Multidomain intervention group

According to the study design, the intervention methods in the present study primarily included exercise training, sleep guidance, meditation training, dietary guidance, and health education. Each community will provide an activity room (about 100 square meters) for our project, and all interventions will be implemented in the community where the subjects live.

In the exercise intervention part, the project team invited geriatrics and physical training experts to develop a six-month advanced exercise training program tailored to the physical characteristics of older adults in China. Resistance training, aerobic exercise, flexibility & balance training, and traditional Chinese exercise were the main components of the exercise program (modified Taijiquan and Baduanjin) (Table 2). Two fixed physical training experts guided all subjects in the multidimensional intervention group through the entire exercise intervention process. The intervention method is offline group intervention with an intervention frequency of three times per week, and each intervention lasts approximately one hour. The following is the main process of exercise intervention (Table 3): [1] pre-exercise mobilization, the coach introduces the course content, understands the status of the subjects, and encourages the subjects to exercise; [2] warm-up exercise, primarily static stretch; [3] formal training courses shall be conducted following the established training plan; [4] relaxation exercise, primarily static stretch; [5] post-exercise stimulation, the coach summarized the training situation, subjects provided feedback on training feelings and questions, and the coach gave positive stimulation and introduced the general content of the next training course.

**Table 2:** Subject’s advanced training intensity level and arrangement (6 months)

Training action package intensity levels	Month					
	Level 1 (L)		Level 2 (M)		Level 3 (H)	
	1	2	3	4	5	6
★ (L)	**√**	**√**				
★ ★		**√**	**√**	**√**		
★ ★ ★				**√**	**√**	
★ ★ ★ ★					**√**	**√**
★ ★ ★ ★ ★ (H)						**√**

Note: Each training package includes Resistance training, aerobic exercise, flexibility & balance training, and traditional Chinese exercise, etc. With the increase of stars, the training intensity is also gradually increasing

**Table 3:** The main process and content of exercise intervention course

Contents	Step 1	Step 2	Step 3	Step 4	Step 5
	Pre-exercise	Warm-up	Formal training	Relaxation exercise	Post-exercise
Mobilization	**√**				
Static stretch		**√**		**√**	
Training package			**√**		
Summary & feedback					**√**

The sleep intervention section mainly relies on community on-site sleep guidance to carry out the intervention. The target participants receive sleep assessment, Q&A, healthy sleep behavior guidance, and feedback. This is the responsibility of the sleep expert, and offline, sleep guidance will be conducted every 1.5 months.

Meditation intervention, with the same frequency as exercise intervention (three times per week). Meditation experts train participants for 15–20 min after each exercise intervention. The meditation expert guided the group members to give feedback and answer questions about the effect of meditation at the end of each complete intervention and pushed the participants to communicate their feelings about meditation.

In the dietary intervention section, according to the characteristics of energy intake/consumption of the older adults in China, and with reference to the “Mediterranean diet” to formulate strategies, nutrition experts are invited to formulate grading factors with “energy level” as the dietary recommendation principle. In each “energy level”, a different diet is assigned, with the goal of “promoting cognitive health” as the overarching goal. The nutrition experts matched specific recipes for the subjects and interpreted them one-to-one based on the participants’ diet scale and diet-related behavior assessment results. Every 1.5 months, nutritional dietary counseling will be provided. Simultaneously, after each offline exercise and meditation intervention, the participants are urged and reminded to eat according to their diet and given positive incentives.

#### Health education intervention group

Community on-site group teaching are used for the health education part of the present study, which covered 24 learning topics (Frequency & form: 1 h each time, once a week for 6 months, fixed time and place) such as scientific exercise, joint pain, healthy sleep, nutritious diet, hypertension, diabetes, cognitive disorders, diseases, and new therapies for older adults. The project team will prepare the paper publicity materials based on the theme of each lecture and distribute the health education materials before each lecture. More than ten doctors were organized to conduct a six-month offline health education intervention course for the target participants in the pilot trial of our study.

#### Non-contact control group

Subjects in this group only performed compliance related maintenance work, such as birthday greeting messages and holiday gifts (such as a bouquet of flowers), in addition to enrollment and exit assessment.

During the implementation of this study, when the subjects feel that the experimental intervention has a high risk to themselves or increases the burden of disease, our team will fully communicate with the participants and give professional advice. If the subjects still insist on withdrawing after full consideration, the research team will respect the requirements of the subjects and make relevant records.

### Assignment of interventions

#### Sequence generation

The randomization of the code is shared by the data platform manager and PI. Whole random process of this study mainly consists of two steps. The first step is to carry out the random assignment work with the community as the basic unit, select 3 communities at random within the main urban area of the city where the sub-center is located, and use the computer generator randomizer method to determine the intervention content of each community. The second step of the random process is to carry out random assignment with individual residents as the basic unit, and the computer generator randomizer method is used to randomly screen all the candidate subjects in each community who have completed all assessments and are willing to participate in this study, finally the final list of subjects in each community is randomly confirmed. The PI will collect the participants’ cell phone number list and community list, and the data platform manager will use the computer generator randomizer method to match each cell phone number with a random code. The PI then distributes the community and participant codes to the community managers, who use them to carry out the sequence generation.

#### Randomization, allocation and concealment

The roles involved in the implementation of the study in each sub-center included PI, data platform manager, participant, community manager (research team), community partner, multidimensional intervention implementation team, health education implementation team, cognitive assessment team, and radiology testing team. A computer generator randomizer method (PI execution) is used to determine the intervention content in each community, and PI is responsible for conducting specific research work in each sub-center. This study could not achieve a perfect “blinding” because the participants are able to clearly perceive the intervention, but we tried to maximize the “isolation” of the intervention/control implementation team in each group to reduce the interaction between the groups.

The intervention/control settings of the three communities in each sub-center are assigned by the platform manager using the computer generator randomizer method to achieve the randomization process in our study.

The PI organizes and contacts all research processes independently, and the data platform manager is not involved in the community effort. All participants in the same community received the same intervention, and subjects in different communities were unaware of each other’s existence. The PI assigns all the research work for their respective communities to three permanent community managers who do not know each other and do not collaborate with the investigator. Community partners are only responsible for research projects in their communities. There is no overlap between the multidimensional intervention and health education teams because they are only responsible for the research in their communities. The cognitive assessment team is primarily composed of temporarily recruited medical students. The team receives homogeneity training from the PI and data platform manager on the use of standards of the cognitive assessment tools and how to use the online platform. The functional MRI test technician of the radiology team only knows the participants` code number and does not participate in any community intervention work except the functional MRI test. The test data file is labeled as “ID + test time”, and PI will retain all data. All cognition-related assessments are collected paperlessly, and community on-site assessment data are directly transmitted to the online research platform (link address: https://ncrcg-brainhealth-1301126429.file.myqcloud.com) to ensure fast and direct data transmission, reducing intermediate transmission processes, and ultimately reduce the risk of contamination.

### Outcomes

Using the gold standard scale as the primary outcomes, the effect of intervention can be evaluated scientifically. At the same time, exercise ability, sleep, nutrition, anxiety, depression and biological indicators are also closely related to cognitive dysfunction, and imaging indicators will be considered as secondary outcomes of this study.

#### Primary outcomes

1. Cognitive function: The assessment methods included the Montreal Cognitive Assessment (MoCA) Scale, the Subjective Cognitive Decline (SCD-Q9) Scale, the Auditory verb learn test-Huashan version (AVLT-H) Scale, the Shape Trails Test (STT-A/B) Scale, the Animal Fluency Test (AFT) Scale, the Boston Naming Test (BNT) Scale, and the Self-Consciousness Scale (SCS).
2. Magnetic Resonance Imaging (MRI): T1, T2, T2flair, Diffusion Weighted Imaging (DWI), Blood Oxygenation Level Dependent (BOLD), and Diffusion Tensor Imaging (DTI).

#### Secondary outcomes

1. Physical function: It primarily includes sarcopenia (muscle mass, muscle strength, and physical function), the Fried Scale, and International Physical Activity Questionnaire Short Form (IPAQ-SF).
2. Sleep: Pittsburgh Sleep Quality Index (PSQI).
3. Nutritional status: the multi-nutritional assessment short form (MNA-SF) and body mass index (BMI).
4. Mood disorders: Patient Health Questionnaire-9 (PHQ-9) Scale, and the Generalized Anxiety Disorder-7 (GAD-7) Scale.
5. The plasticity of multidimensional intervention on cognitive impairment biomarkers: The influence of the multidimensional intervention on cognitive impairment biomarkers was studied by micro-protein post-translational modification, metalomics, metabolomics, transcriptomics, and other technologies and compared with AD molecular markers to determine their relationship with disease severity and outcome. To explore the effects of the multidimensional intervention on brain perfusion, iron content, β-amyloid protein, Tau protein and phosphorylated Tau protein, deacetylase 1, IL-6, IGF-1, nitric oxide, platelet, ubiquitin, C-reactive protein, hemocysteine, and other key indicators, quantitative susceptibility mapping (QSM), structural MRI imaging, fluid attenuated inversion recovery (FLAIR) sequence, amyloid and tau PET were used.

### Data collection and analysis

#### Data collection methods

Cognitive-related professional assessments (Table 4) will be performed on all formally enrolled participants during enrollment and exit, including basic personal information collection, comprehensive assessment of cognitive health, physical condition assessment, functional MRI testing, biological samples testing/ storing, and wearable device monitoring (sports watches).

**Table 4:** Contents list of participants’ enrollment and exit evaluation

Data Type	Dimension	Details/tools
Basic personal information collection	General units	Name, gender, age, ID number, place of birth, length of local residence, local residential address, telephone number (her/himself + relatives), educational background, nationality, language (minority language), and main occupation before 60 years old.
Comprehensive assessment of cognitive health	Cognitive function	MoCA Scale, MMSE Scale, DSST Scale, AVLT-H Scale, STT Scale, AFT Scale, and BNT-30 Scale
	Daily living ability	ADL Scale, and IADL Scale.
	Mental	PHQ-9 Scale, and GAD-7 Scale.
	Sleep	PSQI Scale.
	Nutrition	MNA-SF Scale.
	Physical exercise	IPAQ-SF Scale.
	Sarcopenia	SARC-F Scale.
	Frail	FRAIL Scale.
	Social function information	SSRS Scale.
	Others	Previous disease history, hospitalization history \ Medication status \ diet, drinking, and smoking.
Physical condition assessment	General units	Body mass index, blood pressure, waist-hip ratio, biceps circumference, triceps skinfold thickness, arm span, and calf circumference.
	Sarcopenia	Handgrip strength (Xiangshan EH102), six-meter walk, five-time chair stand test, appendicular skeletal muscle mass and BIA (Inbody 770).
Functional magnetic resonance imaging testing	Cognitive related units	T1, T2, T2flair, DWI, BOLD, DTI.
Biological samples testing/ storing	Informed sample testing information	Blood routine, biochemistry, trace elements, inflammatory factors, hormones, stool, and urine routine.
	Cryopreserved samples (-80 ˚C)	Blood and stool.
Wearable device monitoring (Huawei band6 sports watches)	Sleep data	Total sleep duration, deep sleep duration, and light sleep duration.
	Movement data	Heart rate.

Note: MoCA, Montreal Cognitive Assessment; MMSE, Mini-mental State Examination; DSST, Digit Symbol Substitution Test; AVLT-H, Auditory verb learn test-Huashan; STT, Shape Trails Test; AFT, Animal Fluency Test; BNT, Boston Naming Test; ADL, Activities of Daily Living; IADL, Instrumental Activities of Daily Living; PHQ-9, Patient Health Questionnaire-9; GAD-7, Generalized Anxiety Disorder-7; PSQI, Pittsburgh Sleep Quality Index; MNA-SF, Multi-Nutritional Assessment Short Form; BIA, Bioelectrical Impedance Analysis; IPAQ-SF, International Physical Activity Questionnaire Short Form; SARC-F, Sarcopenia-Five; SSRS, Social Support Rating Scale; DWI, Diffusion Weighted Imaging; BOLD, Blood Oxygenation Level Dependent; DTI, Diffusion Tensor Imaging

#### Statistical methods

To compare the results of cognitive assessment among groups, quantitative data analysis will use IBM SPSS version 21.0 [27] and Python version 3.0 [28] software, relying on the analysis of variance, covariance, and other methods. Mean, standard deviation (SD), median, and range will describe continuous variables. Counting, proportion, the Wilcoxon test, and other methods will be used to analyze categorical variables. Classify and compare the normal distribution, homogeneity of variance and other data features of each variable in all the data, preliminarily screen variables with statistical differences, and combine professional knowledge to use these variables as alternative variables for subsequent comparative analysis and model training. Graphical summaries such as boxplots, case profiles, and labeled dispersion maps will be used to compare the distribution of intervention variables in each group and participant characteristics between groups. A linear mixed model will be used to simulate longitudinal changes in primary responses between groups, allowing for missing data (assuming data is missing at random) and within-subject correlations for repeated measures over time, with the model adjusting for baseline response variables and other covariates as needed. The effectiveness analysis (Full Analysis Set, Per Protocol Set) will be performed to assess the overall effect of the intervention.

Using data analysis techniques such as regression, chi-square, T-test, rank-sum test, normality test, decision tree, support vector machines, XGBoost, CATBoost and artificial intelligence deep learning, this study will rely on multi-modal data, take training effect and cognitive improvement as label values, and build a prediction and intervention model of mild cognitive dysfunction supported by artificial intelligence technology. Simultaneously, our team will apply explicable machine learning theory to explain the variables in the model and try to create new ideas for the basic research direction.

## Discussion

### Adherence strategy

The compliance strategy starts with determining how to improve the adherence of participants and the intervention implementers (community managers).

Before the implementation of the project, the study group conducted a series of compliance tests using a series of combination methods to include participants with high compliance in the study. Community managers must not only have a certain level of medical professionalism, but they must also be emotionally intelligent, agreeable, and able to maintain a good and harmonious relationship with participants. During the study, core information on health behaviors will be published daily in the online WeChat group to increase crowd adherence. The research team will prepare appropriate holiday gifts for the participants (inexpensive but meaningful, such as a flower for all subjects on Mother’s Day, etc.). If participants raise questions in the online WeChat group, the community manager should respond in time (generally within 10 min) and provide positive feedback. After the group intervention, the community manager instructed the participants to communicate with one another to increase the bonding effect of the intervention at the level of peer effect. For participants who completed 100%, 90%, and 80% of the intervention content, various reward strategies, such as “fast track” appointments and “face-to-face” expert consultations, will be provided. The community manager will be responsible for counting the participation times of the participants and publishing the times and ranking situation in the WeChat group online every week (usually on Friday).

Although the outcome of the study is very important, the characteristics of the staff in the implementation process, such as the identification of the values of the study, the degree of implementation of the research protocol, and other factors, are important influencing factors that determine the success of the study. Each group requires a consistent community manager who cannot be changed frequently.

All subjects are not allowed to participate in other lifestyle intervention or drug intervention trials during this study period. During the study period, if a subject does participate in another lifestyle intervention or drug trial, we will inform them that although they can continue to participate in the program, they will not be provided with follow-up evaluations and related tests (fMRI, blood tests, etc.) and their personal data will be disregarded and not used in the analysis.

### Quality control

Quality control methods (online + offline) included regular study progress meetings (WCHSCU & sub-centers Principal Investigator (PI)), methodology training, “regular flight supervision” (The research team will travel from Chengdu to the city where the sub-center is located to supervise the ongoing research, and the supervision frequency is once every 6–8 months to ensure the quality of the research), experience sharing, and exchange discussion (multidimensional intervention group, health education group, and non-contact control group are carried out independently). The core points for quality control of each sub-center are as follows.

#### Participants and communities

The main goal of subject-level quality control is to screen the subjects according to the eligibility criteria, implement the project strictly according to the research plan, take the initiative to understand the feelings, questions, and appeals of the participants during their participation in our program, and establish a good relationship between the community research manager and the participants on the premise of not interfering with the whole research.

The distance between any two research communities should be greater than 5 km to avoid the influence of contamination factors on the research. In the present study, community partners must meet the following criteria: [1] have independent office areas (such as non-governmental organizations or property service centers) in the study community; [2] have an excellent community-friendly foundation and have carried out resident activities in the community; and [3] cooperate with the research team to provide support for publicity and intervention sites within the community.

#### Groups perform standards and quality control

During the intervention phase, community managers and the PI will be jointly responsible for quality control. Community managers will be responsible for communicating with the intervention implementation team, community partners, and participants to confirm the site, time, and staffing arrangements related to the intervention to meet the objective conditions for implementing the study protocol. The following are the key quality control points in each group.

Multidimensional intervention group [1]. All intervention activities should be completed in the community of the participants [2]. The exercise intervention will be taught alternately by two coaches, with no additional coaches added during the intervention. The exercise training will be taught according to the training schedule, and the training plan will not be altered during the course. After each training, the coach will give the participants positive motivation and encouragement and collect the subjects’ training feedback. To guide and correct the training movement standards, the number of participants in each offline training intervention cannot exceed 30; if there are > 30 participants, two coaches must be present simultaneously to ensure that each participant receives enough attention. The weekly intervention time should be fixed and remain unchanged. To achieve adequate training supervision, the training coach will use the sports watch to monitor the heart rate and other indicators of the participants [3]. Meditation intervention requires the preparation of meditation tasks (instructions and background music) in advance, and the same meditation task cannot be repeated within a month. The meditation training began within 5–10 min of the end of the exercise intervention.

The participants will be reminded not to sleep before meditation. After completing the meditation, it is necessary to guide the subjects to communicate with one another about their meditation feelings, which is beneficial to developing a social support network in the intervention group. At the end of all courses, feedback from the participants will be collected. Participants will be encouraged to practice self-training meditation [4]. The quality control of the dietary intervention focused on supervising and encouraging subjects to eat according to the recipes. Community managers will be responsible for encouraging participants to send photos of three meals daily to monitor whether they eat according to the recommended recipes. To avoid the awkward situation of different recipes in one family, the same family members’ recipes are consistent. Community managers will provide one-on-one supervision and encouragement to participants who do not post food photos [5]. To improve the sleep behavior of the participants, the community managers regularly pushed popular science articles on the topic of “sleep health” in the online WeChat group (once a week). There will be a free community on-site consultation with a sleep specialist. Instead of traditional passive knowledge dissemination (such as non-interactive “PPT teaching”), offline health lectures take the form of interactive teaching.

Health education group [1]. All intervention activities should be completed in the community of the participants [2]. The six-month health education course is completed by six doctors, which facilitates PI supervision of teaching quality and the maintenance of subject compliance [3]. The weekly course time is fixed (2 to 3 pm every Wednesday) and cannot be changed [4]. Every Monday, community managers remind the details of course time, place, teaching topic, and teaching teacher information in the online WeChat group [5]. One-on-one communication will be conducted to encourage participants who have failed to come or requested leave for two consecutive times to avoid dropping out [6]. During traditional Chinese festivals, small gifts will be prepared according to the festival atmosphere for participants to improve their compliance and recognition of this study.

Non-contact control group [1]. whether to prepare small gifts for participants during traditional Chinese festivals; [2] The number of subjects in this group should be increased appropriately (by about 10%) to cope with the dropout due to uncontrollable factors.

#### Data acquisition

The questionnaire assessment and equipment testing are important quality control links in the assessment section.

Only evaluators who pass the assessment can enter the community to assess the older-adult residents. The data platform manager completes the setup of online evaluation tools, including content construction, cell restriction, logic jump setting, and review, before entering the community to conduct the evaluation. The questionnaire assessment process of each community resident will be completed independently by a fixed questionnaire assessor (trained student volunteers), and the evaluation method will be a community on-site assessment. And resident must answer all questions, and no family members or other people are permitted to do so. The current students will be hired as the “third independent person” telephone return visitors the day after the on-site assessment, and 20% of the evaluated participants on the day are randomly selected to conduct a second telephone inquiry and check on some core questions in the questionnaire (the daily check content is randomly generated, and the telephone return visit time is approximately 1–3 min).

Equipment quality control uses unified brand instruments and equipment to measure suitable indicators. Standard operating procedures for instruments and equipment can help different operators follow the same procedures. Before using the instrument, it should be debugged and corrected, and records should be kept for future reference. In principle, it is recommended that all instruments be operated by fixed operators or that uniform training and assessment be provided. For data traceability, all data generated by instruments and equipment must be accurately recorded and backed up. The data copy principle is “today’s data, and today’s copy”, which is stored in a special mobile hard disk owned by PI and backed up to the data platform manager.

In the face of missing data due to uncontrollable factors, the following steps should be taken to trace, retest or deal with it to minimize the loss: [1] Discuss the reasons for missing data and potential remedies within the project team; [2] Attempts to find historical data or contact subjects for data tracing or retesting; [3] If it is really impossible to make up for the missing data, the project team members shall discuss and determine whether to eliminate the data or use statistical methods to make up for it.

#### Biological sample

Before biological specimen collection and detection, all participants must sign informed consent forms. The contents of verification in the collection process included whether the blood samples labeling information is accurate and clear, whether the temporary storage environment after collection is 4–6 ˚C, and whether the collection is centrifuged within 2 h. The contents of verification during the specimen transfer process included whether the transfer temperature is 4–6 ˚C, whether the specimen is damaged during the transfer, and whether there is transfer record information. The verification content of the specimen detection process included whether 3–5% of the samples are repeated detection and result comparison reports and whether the test data are properly stored. The inspection contents during specimen storage included the list of specimens in and out of storage records, specimen storage information (preservation temperature, type, location, and basic information), and pretreatment process record information before specimen freezing (-80 ˚C).

### Monitoring

#### Data monitoring, confidentiality and management

NCRCG and WCHSCU has a comprehensive data management system (including collection, quality control, cleaning, archiving, application, use, and other management methods), and all data generated in the present study will be managed in accordance with this method. In order to ensure the safety and compliance of data use, a data monitoring committee (DMC) had been set up, which is mainly responsible for data authenticity and security, data quality control, original research data preservation, data application and use, etc. The members of the committee are mainly composed of PI, data platform managers, etc. DCM is independent from the sponsor and competing interests. The following are the specific data security links.

Subjective data assessment is used online platform (https://ncrcg-brainhealth-1301126429.file.myqcloud.com) to improve the collection. The platform is composed of independent research and development by the study team. The server is managed by dedicated personnel and is located in the NCRCG (5th Floor, Building 1, Jitaian Center, 17 Gaopeng Avenue, Wuhou District, Chengdu City, Sichuan Province, China).

The PI and platform managers are jointly responsible for exporting specific data, which are uniformly stored in the server and backed up in the designed mobile hard disk for future reference and verification.

Data from wearable devices (such as sports watches) can be transmitted directly to the platform, ensuring data security and improving data transmission efficiency.

The online data system of our study by the managers responsible for the platform, a platform which can realize the function of the different roles account authorization, such as community manager can see the assessment tools and data results of each subject, independent assessors could only see the assessment tools, the set of such functions, and to some extent to ensure the data security and controllability.

#### Harms and safety

The safety of participants is the top priority of the present study. The collection and feedback mechanism of subjects’ safety problems mainly consists of 5 steps: step 1 is for community manager to collect information; step 2 is the community manager will report the problem to PI; step 3 is for PI to decide whether it is necessary to convene a medical expert group to conduct potential analysis of the problem and discuss countermeasures; step 5 is for feedback to subjects themselves. The following are some potential risks and countermeasures:

##### Potential safety risk I

Direct accident risks, such as car accidents and falls, may occur between the participants’ journey to/from their homes and the offline intervention scenario.

##### Solution I

A community intervention site is established, and only permanent community residents can participate in this study. To deal with the potential accident risk of participants between home and the training site, the study team will purchase accident insurance for each participant.

##### Potential safety risk II

During the exercise intervention, participants may sustain unpredictable injuries due to non-standard movements, such as muscle strain, fracture, and accidental fall.

##### Solution II

A progressive exercise training program appropriate for older adults will be implemented [29], with participants guided by professional coaches. First, they will be taught the movement norms and key points. Then, based on the progressive training program and the participant feedback, older adults will be given systematic and progressive exercise intervention training. Purchase clinical trial insurance for each participant to cover any unexpected risk from the intervention.

##### Potential safety risk III

Potential risks during the data analysis and biological specimen collection processes, such as extensive bruising on the skin surface after blood drawing, dizziness, hypoglycemia, falling, and other emergencies.

##### Solution III

Provide systematic training for the biological specimen collection personnel, such as informing the blood drawing nurse to recommend the proper pressing method for participants and teaching all staff how to recognize hypoglycemic symptoms. We are training all staff on emergency response plans. Assign special personnel to be responsible for the risk investigation work of the field assessment based on the risk investigation list of epidemiological field assessment.

##### Potential safety risk IV

During functional MRI examination of the head, participants may experience claustrophobia.

##### Solution IV

Functional MRI technicians and community managers must repeatedly confirm with participants whether they have claustrophobia before functional MRI examination. Simultaneously, they should pay close attention to the status of participants during the investigation. If the older adult has claustrophobia, our team members should provide scientific and reasonable advice while respecting the subjects’ decision to continue the examination.

##### Potential safety Risk V

Based on our assessment, the participant is suspected of having an acute disease.

##### Solution V

Provide participants timely feedback on test results, professional medical advice, and potential treatment strategies.

#### Auditing

NCRCG employs an independent auditor team to conduct independent audits of the research process (frequency: once every six months), including but not limited to research funding, original research data verification, on-site supervision verification, etc.

### Future plan

The key of this study is to construct an early multidimensional intervention strategy for residents with MCI in the community based on the premise of “Chinese population characteristics”. The following aspects will be the focus of the next step.

1. Summarize the pilot study experience. Our team will summarize the problems and experiences in the pilot trial, including the research process, problems and solutions, intervention tips for each group, compliance maintenance, study details implementation, and community random screening. Furthermore, form a set of replicable research work manuals for other sub-centers to use as a reference.
2. Increase the sample size of the study. Due to the vast territory of China, each region has its characteristics of life and behavior. To develop a multidimensional intervention strategy for MCI patients in the community based on “Chinese population characteristics”, it is necessary to verify it in a larger sample population to carry out more diverse intervention logic and content. Therefore, the present study will be validated in a more diverse population sample.
3. Biological specimen detection and mechanism research. Our team will examine the collected biological specimens, including special biomarkers, metabolomics, and proteomics of frozen blood samples. The frozen stool samples will be analyzed for intestinal microbiota. To investigate the influencing factors and mechanisms of intervention measures on cognitive impairment using differences in biological samples results based on the subjects’ controls.
4. According to the intervention strategy of this study, we tried to build an online cognitive intervention platform, establish an online cognitive health promotion tool (individualized plan) in line with the characteristics of the Chinese population, and translate the research results.

### Protocol amendments

The research program is an important support for this study, and also affects the interests and direction of subjects and PI. Changes in the research program, Including changes of study objectives, study design, patient population, sample sizes, study procedures, or significant administrative aspects are reported to the researcher, the Ethics Committee, the study branch, the subject, etc.

### Protocol version

Issue date: 2023 Jan 20.

Protocol amendment number: 2.0.

Authors: XW, LW, BD.

### Disclaimer

Our sources of funding had no role in the design of our research, and will not be any impact on data collection, analysis, writing and decision to submit or publish the research results.

### Patient and public involvement statement

It was not appropriate or possible to involve patients or the public in the design, or conduct, or reporting, or dissemination plans of our research.

### Electronic supplementary material

Below is the link to the electronic supplementary material.

Supplementary Material 1

## Data Availability

Researchers interested in the Cognitive Impairment Intervention are welcome to visit our website (http://www.wchscu.cn/scientific/clinical/platform/55440.html) for more information. If you have any questions or would like to collaborate, please contact us via email (hxncrcg@163.com).

## Abbreviations

MCI: Mild Cognitive Impairment
RCT: Randomized Controlled Trial
AD: Alzheimer’s disease
WHO: World Health Organization
AAN: American Academy of Neurology
MIND: Mediterranean-Dash diet Intervention for Neurodegenerative Delay
MAPT: Multidomain Alzheimer Preventive Trial
BMI: Body Mass Index
SPIRIT: Standard Protocol Items:Recommendations for Interventional Trials
WCHSCU: West China Hospital of Sichuan University
NCRCG: National Clinical Research Center for Geriatrics
MoCA: Montreal Cognitive Assessment
MMSE: Mini-mental State Examination
PAR-Q: Physical Activity Readiness Questionnaire
FMRI: Functional Magnetic Resonance Imaging
PI: Principal Investigator
SCD-Q9: Subjective Cognitive Decline
AVLT-H: Auditory verb learn test-Huashan version
STT: Shape Trails Test
AFT: Animal Fluency Test
BNT: Boston Naming Test
SCS: Self-Consciousness Scale
DWI: Diffusion Weighted Imaging
BOLD: Blood Oxygenation Level Dependent
DTI: Diffusion Tensor Imaging
IPAQ-SF: International Physical Activity Questionnaire Short Form
PSQI: Pittsburgh Sleep Quality Index
MNA-SF: Multi-nutritional Assessment Short Form
PHQ-9: Patient Health Questionnaire-9
GAD-7: Generalized Anxiety Disorder-7
QSM: Quantitative Susceptibility Mapping
FLAIR: Fluid Attenuated Inversion Recovery
SD: Standard Deviation

## References

[1] Breijyeh Z, Karaman R. Comprehensive Review on Alzheimer’s Disease: causes and treatment. Molecules. 2020;25(24). 10.3390/molecules25245789 PMC7764106 33302541

[2] DeTure MA, Dickson DW (2019). The neuropathological diagnosis of Alzheimer’s disease. Mol Neurodegener.

[3] Ren R, Qi J, Lin S, Liu X, Yin P, Wang Z (2022). The China Alzheimer Report 2022. Gen Psychiatr.

[4] Lopez OL, Kuller LH (2019). Epidemiology of aging and associated cognitive disorders: prevalence and incidence of Alzheimer’s disease and other dementias. Handb Clin Neurol.

[5] Rasmussen J, Langerman H (2019). Alzheimer’s Disease - Why we need early diagnosis. Degener Neurol Neuromuscul Dis.

[6] Salzman T, Sarquis-Adamson Y, Son S, Montero-Odasso M, Fraser S (2022). Associations of Multidomain interventions with improvements in cognition in mild cognitive impairment: a systematic review and Meta-analysis. JAMA Netw Open.

[7] Jia J, Zhou A, Wei C, Jia X, Wang F, Li F (2014). The prevalence of mild cognitive impairment and its etiological subtypes in elderly chinese. Alzheimers Dement.

[8] Sherman DS, Mauser J, Nuno M, Sherzai D (2017). The efficacy of cognitive intervention in mild cognitive impairment (MCI): a Meta-analysis of outcomes on neuropsychological measures. Neuropsychol Rev.

[9] Petersen RC, Lopez O, Armstrong MJ, Getchius TSD, Ganguli M, Gloss D (2018). Practice guideline update summary: mild cognitive impairment: report of the Guideline Development, Dissemination, and implementation Subcommittee of the American Academy of Neurology. Neurology.

[10] Huang X, Zhao X, Li B, Cai Y, Zhang S, Wan Q (2022). Comparative efficacy of various exercise interventions on cognitive function in patients with mild cognitive impairment or dementia: a systematic review and network meta-analysis. J Sport Health Sci.

[11] Arjmand G, Abbas-Zadeh M, Eftekhari MH (2022). Effect of MIND diet intervention on cognitive performance and brain structure in healthy obese women: a randomized controlled trial. Sci Rep.

[12] Cherian L, Wang Y, Fakuda K, Leurgans S, Aggarwal N, Morris M (2019). Mediterranean-Dash intervention for neurodegenerative Delay (MIND) Diet slows cognitive decline after stroke. J Prev Alzheimers Dis.

[13] Liu X, Morris MC, Dhana K, Ventrelle J, Johnson K, Bishop L (2021). Mediterranean-DASH intervention for neurodegenerative Delay (MIND) study: Rationale, design and baseline characteristics of a randomized control trial of the MIND diet on cognitive decline. Contemp Clin Trials.

[14] van den Brink AC, Brouwer-Brolsma EM, Berendsen AAM, van de Rest O (2019). The Mediterranean, Dietary Approaches to stop hypertension (DASH), and Mediterranean-DASH intervention for neurodegenerative Delay (MIND) diets are Associated with Less Cognitive decline and a lower risk of Alzheimer’s Disease-A Review. Adv Nutr.

[15] Winer JR, Deters KD, Kennedy G, Jin M, Goldstein-Piekarski A, Poston KL (2021). Association of short and long sleep duration with amyloid-beta burden and cognition in aging. JAMA Neurol.

[16] Zeidan F, Johnson SK, Diamond BJ, David Z, Goolkasian P (2010). Mindfulness meditation improves cognition: evidence of brief mental training. Conscious Cogn.

[17] Rosenberg A, Mangialasche F, Ngandu T, Solomon A, Kivipelto M (2020). Multidomain Interventions to prevent cognitive impairment, Alzheimer’s Disease, and dementia: from FINGER to world-wide FINGERS. J Prev Alzheimers Dis.

[18] Dang A, Arora D, Rane P (2020). Role of digital therapeutics and the changing future of healthcare. J Family Med Prim Care.

[19] Biddle GJH, Edwardson CL, Rowlands AV, Davies MJ, Bodicoat DH, Hardeman W (2019). Differences in objectively measured physical activity and sedentary behaviour between white Europeans and south Asians recruited from primary care: cross-sectional analysis of the PROPELS trial. BMC Public Health.

[20] Xin J, Du M, Gu D, Jiang K, Wang M, Jin M (2023). Risk assessment for colorectal cancer via polygenic risk score and lifestyle exposure: a large-scale association study of east asian and european populations. Genome Med.

[21] Razieh C, Khunti K, Davies MJ, Edwardson CL, Henson J, Darko N (2019). Association of depression and anxiety with clinical, sociodemographic, lifestyle and environmental factors in south asian and white european individuals at high risk of diabetes. Diabet Medicine: J Br Diabet Association.

[22] Li Z, Jongbloed L, Dean E (2014). Stroke-related knowledge, beliefs, and behaviours of chinese and european canadians: implications for physical therapists. Physiotherapy Can Physiotherapie Can.

[23] Dai Q, Su H, Zhou Z, Li C, Zou J, Zhou Y et al. Psychometric evaluation of the chinese version of mild cognitive impairment questionnaire among older adults with mild cognitive impairment. Int J Environ Res Public Health. 2022;20(1). 10.3390/ijerph20010498 PMC9819359 36612819

[24] Ciesielska N, Sokolowski R, Mazur E, Podhorecka M, Polak-Szabela A, Kedziora-Kornatowska K (2016). Is the Montreal Cognitive Assessment (MoCA) test better suited than the Mini-Mental State Examination (MMSE) in mild cognitive impairment (MCI) detection among people aged over 60? Meta-analysis. Psychiatr Pol.

[25] Hao L, Wang X, Zhang L, Xing Y, Guo Q, Hu X (2017). Prevalence, risk factors, and Complaints Screening Tool Exploration of Subjective Cognitive decline in a large cohort of the Chinese Population. J Alzheimers Dis.

[26] Rutterford C, Copas A, Eldridge S (2015). Methods for sample size determination in cluster randomized trials. Int J Epidemiol.

[27] Ma K, Shi Y, He J, Teng X, Wang R, Wang G (2022). The effect of Bushen Culuan Decoction on anovulatory infertile women among 6 different diseases: a study protocol for a randomized, double-blinded, positively controlled, adaptive multicenter clinical trial. Trials.

[28] Weiss CJ. Visualizing protein big data using Python and Jupyter notebooks. Biochemistry and molecular biology education: a bimonthly publication of the International Union of Biochemistry and Molecular Biology. 2022;50(5):431-6. 10.1002/bmb.21621 35403803

[29] Izquierdo M, Merchant RA, Morley JE, Anker SD, Aprahamian I, Arai H (2021). International Exercise Recommendations in older adults (ICFSR): Expert Consensus Guidelines. J Nutr Health Aging.

